# The TraDIS toolkit: sequencing and analysis for dense transposon mutant libraries

**DOI:** 10.1093/bioinformatics/btw022

**Published:** 2016-01-21

**Authors:** Lars Barquist, Matthew Mayho, Carla Cummins, Amy K. Cain, Christine J. Boinett, Andrew J. Page, Gemma C. Langridge, Michael A. Quail, Jacqueline A. Keane, Julian Parkhill

**Affiliations:** ^1^; ^2^

## Abstract

**Summary:** Transposon insertion sequencing is a high-throughput technique for assaying large libraries of otherwise isogenic transposon mutants providing insight into gene essentiality, gene function and genetic interactions. We previously developed the Transposon Directed Insertion Sequencing (TraDIS) protocol for this purpose, which utilizes shearing of genomic DNA followed by specific PCR amplification of transposon-containing fragments and Illumina sequencing. Here we describe an optimized high-yield library preparation and sequencing protocol for TraDIS experiments and a novel software pipeline for analysis of the resulting data. The Bio-Tradis analysis pipeline is implemented as an extensible Perl library which can either be used as is, or as a basis for the development of more advanced analysis tools. This article can serve as a general reference for the application of the TraDIS methodology.

**Availability and implementation:** The optimized sequencing protocol is included as supplementary information. The Bio-Tradis analysis pipeline is available under a GPL license at https://github.com/sanger-pathogens/Bio-Tradis

**Contact:** parkhill@sanger.ac.uk

**Supplementary information:** Supplementary data are available at *Bioinformatics* online.

## 1 Introduction

Steady improvements in high-throughput sequencing technologies have resulted in an increasing number of sequenced bacterial genomes, revealing extensive genetic diversity both within and between species. Associated sequencing-based technologies, such as RNA-seq, ChIP-seq and RIP-seq provide insight into the effects of this variation on gene expression and regulation; however, none provides direct information on cell survival, and hence how this genetic variation may impact the fitness of the bacterium (Gray *et al.*, 2015). Transposon insertion sequencing (TIS) bridges this gap between sequence and fitness by allowing for direct measurement of survival dynamics within a population of single transposon mutants, by using sequencing reads flanking transposon insertions as a read-out of mutant frequency within the population (Barquist *et al.*, 2013a; Van Opijnen and Camilli, 2013). We previously developed a method for this purpose, called Transposon Directed Insertion Sequencing (TraDIS; (Langridge *et al.*, 2009). TraDIS uses fragmentation of genomic DNA followed by specific PCR amplification of transposon-containing fragments to selectively enrich for transposon-flanking sequences, and can be adapted for any transposon of interest through a simple redesign of sequencing primers. TraDIS has since been applied to a variety of target organisms and transposons in a wide variety of both *in vivo* and *in vitro* growth conditions. These include Tn*5*-based libraries in *Salmonella* (Barquist *et al.*, 2013b; Chaudhuri et al., 2013; Langridge *et al.*, 2009) and *Escherichia* (Dziva et al., 2013; Eckert *et al.*, 2011) and Mariner-based libraries in *Clostridia* (Dembek *et al.*, 2015) and *Mycobacteria* (Weerdenburg *et al.*, 2015).

## 2 Library preparation and sequencing

We have made a number of refinements to the TraDIS sequencing protocol since its initial publication (Langridge *et al.*, 2009), described in more detail in the supplement. We have redesigned TraDIS adapters and primers using a splinkerette approach (Devon *et al.*, 1995; Rad *et al.*, 2015; Uren *et al.*, 2009), which increases enrichment of genuine transposon-chromosome junctions by preventing hybridization of the reverse primer until the transposon-specific forward primer has generated a complementary strand. We have substituted a magnetic bead-based fragment size selection for gel-based size selection to increase yield and allow for easier automation (Bronner *et al.*, 2013). Finally, we have substituted Kapa Hifi DNA polymerase for Taq polymerase, as this enzyme has been shown to have minimal amplification biases (Quail *et al.*, 2012), and reduced the number of cycles of PCR amplification to provide a more accurate representation of input.

TraDIS sequencing primers are designed to begin sequencing within the transposon sequence, so as to provide a short 8–10 base ‘transposon tag’ at the beginning of each read to verify that each read originates from a genuine transposon-chromosome junction. This poses a challenge for Illumina sequencing machines, as the base-calling algorithms assume a complex sample for the purposes of calibration. We have developed HiSeq and MiSeq recipes that use ‘dark cycles’ during which chemistry is run but no imaging is performed to read through this transposon tag, before imaged sequencing commences on the complex chromosomal DNA (see supplement). Once the first read is completed, the DNA is denatured and the transposon-specific sequencing primer is re-annealed for a separate short 10–12 cycle transposon read. This requires a PhiX (or other complex library) spike-in of 5–10% to prevent sequencing failure due to a lack of fluorescence in some channels. Using this protocol we routinely achieve results of > 90% of sequencing reads both containing an intact transposon tag and mapping uniquely to the source genome. We have applied this method to Tn*5-*, Tn*917-*, *Himar1-* and Mu-based mutant libraries, and it should be adaptable to any transposon of interest assuming a suitable priming site exists (see supplement for design parameter details).

## 3 The Bio-Tradis analysis pipeline

To support the use of this improved TraDIS protocol, we have developed a portable processing and analysis pipeline implemented in the Perl and R languages. The functionality provided is similar to that in other recently published TIS analysis pipelines (DeJesus *et al.*, 2015; Solaimanpour *et al.*, 2015), however our command-line driven approach has been designed with a production environment in mind, where many sequencing libraries may be processed simultaneously. We provide tools for each step of analysis from the raw unaligned fastq files produced by the sequencer, through to predictions of gene essentiality and fitness effects. The main pipeline script, **bacteria_tradis**, filters reads in fastq format for transposon tags, removes these tags, then maps the modified reads using the SMALT short read mapper (https://www.sanger.ac.uk/resources/software/smalt/), with support for multiple contigs and/or replicons, such as plasmids. Default k-mer, step size and percent identity parameters are set depending on input read length, though these can be manually specified by the user. The mapped bam file is then processed to produce plot files, containing insertion counts per nucleotide, suitable for visualization in the Artemis genome browser (Carver *et al.*, 2012) and for further analysis. The mapping, processing, and data manipulation steps are implemented as self-contained Perl modules that could be easily used as a foundation for the development of more sophisticated analyses.

Additional scripts are provided to process these plot files in conjunction with genome annotations in EMBL-Bank format to produce annotated tab-delimited files containing various statistics including read counts and unique insertion sites per gene. Two basic analysis scripts for this gene-level data written in R are available. One, **tradis_essentiality.R**, produces predictions of gene essentiality within a high-density transposon library based on the empirically observed bimodal distribution of insertion sites over genes when normalized for gene length (Barquist *et al.*, 2013b; Langridge *et al.*, 2009). The second, **tradis_comparisons.R**, applies the edgeR package (Robinson *et al.*, 2010) to identify significant differences in read counts, and hence mutant frequencies, between experimental conditions (Dembek *et al.*, 2015) providing insight into the relative contribution of all mutagenized genes to fitness under the assayed condition.

## 4 Summary

We have described recent refinements to the TraDIS method for the sequencing and analysis of dense transposon libraries. These include an optimized sequencing protocol, and processing and analysis tools that can rapidly provide insight into the contribution of genomic regions to organismal fitness. It is our hope that making these tools more accessible will accelerate their application to an ever wider variety of bacteria and experimental conditions.

## Supplementary Material

Supplementary Data
