# Supplementary material for: The TraDIS toolkit: sequencing and analysis for dense transposon mutant libraries
Source: Bioinformatics. 2016 Jan 21;32(7):1109–11. doi: 10.1093/bioinformatics/btw022 (PMC4896371; doi:10.1093/bioinformatics/btw022)
Supplement: Supplementary Data [file supp_32_7_1109__index.html]

The TraDIS toolkit: sequencing and analysis for dense transposon mutant libraries — The TraDIS toolkit: sequencing and analysis for dense transposon mutant libraries — Supplementary Data 

# The TraDIS toolkit: sequencing and analysis for dense transposon mutant libraries

## Supplementary Data

files

- Supplementary Data - pdf file
- Supplementary Data - pdf file
