# Supplementary material for: The TraDIS toolkit: sequencing and analysis for dense transposon mutant libraries
Source: Bioinformatics. 2016 Jan 21;32(7):1109–11. doi: 10.1093/bioinformatics/btw022 (PMC4896371; doi:10.1093/bioinformatics/btw022)
Supplement: Supplementary Data [file supp_btw022_BioTraDISTutorial.pdf]

Using the Bio::TraDIS pipeline

Lars Barquist (contact: lars.barquist@uni-wuerzburg.de)

For installation instructions see the README file in the root directory of the distribution. This tutorial provides an example of mapping a library with the Bio:TraDIS pipeline, along with troubleshooting and quality control advice. It will take 2 - 3 hours to complete, and requires ~3 gigabytes of hard disk space.

Introduction

The Bio::TraDIS pipeline provides software utilities for the processing, mapping, and analysis of transposon insertion sequencing data. The pipeline was designed with the data from the TraDIS sequencing protocol in mind (described in Barquist *et al*., 20XX), but should work with a variety of transposon insertion sequencing protocols as long as they produce data in the expected format.

The core of the pipeline is implemented in the `bacteria_tradis` script, which performs read mapping using `smalt` and insertion quantification. This script assumes that your data is in (possibly gzipped) fastq format, with transposon tags attached. E.g. the sequence of your reads should look like:

TTTTTTTTTCCCCCCCCCCCCCCCCCCCCCCCCCCCCCCCCC

Where T's represent transposon sequence, and C's represent flanking chromosomal sequence. If you have used the TraDIS dark cycle protocol, your "transposon tag" and chromosomal read will be in separate reads. These can be concatenated by running the `add_tradis_tags` script on an unaligned BAM file. The Picard tools may be helpful in performing any necessary file conversion operations: http://broadinstitute.github.io/picard/

`bacteria_tradis` outputs so-called plot files in addition to aligned bam files, containing one row per nucleotide in the reference genomic sequence with two columns, one for each strand. These can then be processed to provide gene-level statistics that can be used for further analysis.

As an example this tutorial will use a sequencing run of a *Salmonella enterica* serovar Typhimurium SL1344 mutant library containing an estimated 1 million mutants that we generated in optimizing the TraDIS protocol, which is publicly available from the European Nucleotide Archive (ENA) at http://www.ebi.ac.uk/ena/data/view/ERR541286.

Downloading and preprocessing the data

First we will need the reference genome and annotation for *S.* Typhimurium SL1344. This strain contains four replicons consisting of one chromosome (ENA accession FQ312003) and three plasmids (HE654724, HE654725, HE654726). We will need both embl format annotations and the fasta sequence files for analysis. These can be retrieved through the ENA web interface. Alternatively, they can be downloaded at the command-line using curl with the following commands (note I use `$>` to refer to the command prompt throughout):

```
$> curl
"http://www.ebi.ac.uk/ena/data/view/FQ312003&display=txt&expanded=true
" > FQ312003.embl
$> curl
"http://www.ebi.ac.uk/ena/data/view/HE654724&display=txt&expanded=true
" > HE654724.embl
$> curl
"http://www.ebi.ac.uk/ena/data/view/HE654725&display=txt&expanded=true
" > HE654725.embl
$> curl
"http://www.ebi.ac.uk/ena/data/view/HE654726&display=txt&expanded=true
" > HE654726.embl

$> curl "http://www.ebi.ac.uk/ena/data/view/FQ312003&display=fasta" >
FQ312003.fasta
$> curl "http://www.ebi.ac.uk/ena/data/view/HE654724&display=fasta" >
HE654724.fasta
$> curl "http://www.ebi.ac.uk/ena/data/view/HE654725&display=fasta" >
HE654725.fasta
$> curl "http://www.ebi.ac.uk/ena/data/view/HE654726&display=fasta" >
HE654726.fasta
```

The fasta files can then be concatenated into one reference fasta file using the command:

```
$> cat *.fasta > ref.fasta
```

We will use TraDIS samples from one lane of sequencing of an “input” library for the purposes of this tutorial, accession number ERR541286. This can be downloaded from

http://www.ebi.ac.uk/ena/data/view/ERR541286

The file we require are the submitted BAM files under the column “Submitted files (ftp)”. This will result in the download of two files, 12418_1#10.bam. Alternatively, this can be directly downloaded at the command line using `curl`:

```
$> curl
ftp://ftp.sra.ebi.ac.uk/vol1/ERA320/ERA320707/bam/12418_1%2310.bam >
12418_1#10.bam
```

The BAM file downloaded from the ENA has both transposon tags and chromosomal reads for each sequence, however these are stored as two separate reads. For further processing we need to concatenate these reads. This can be done with the script `add_tradis_tags`:

```
$> add_tradis_tags -b 12418_1#10.bam -o 12418_1#10.tr.bam
```

-b specifies the input BAM file, and -o the output BAM file. We then convert these to gzipped FastQ files for further processing and mapping using samtools:

```
$> samtools bam2fq 12418_1#10.tr.bam | gzip > 12418_1#10.fq.gz
```

A note on QC and read trimming

While we will not go into detail on this here, normally one would want to perform quality control if this were newly produced data, to get a first look at whether the sequencing has worked properly. For this purpose we recommend FastQC:

http://www.bioinformatics.babraham.ac.uk/projects/fastqc/

It is well worth spending time to read through the detailed documentation provided with FastQC. Note that due to the nature of TraDIS libraries they will always fail many of the standard sequencing quality checks (e.g. per base sequence content, kmer content, sequence duplication levels), but the per base sequence quality and reports on adapter contamination can be valuable.

If there is evidence for declining read quality towards the end of reads, or if there is significant adapter contamination, read trimming may help to rescue a low quality run. PRINSEQ (http://prinseq.sourceforge.net/) is well suited to this purpose, as it allows for trimming selectively from the right end of the read; this is important, as we want to keep the transposon tag at the left end intact.

Read processing and mapping

Now that our sequencing runs are in FastQ format, we are ready to run the `bacteria_tradis` mapping pipeline. Running `bacteria_tradis` on the command line with no options will give a list of parameters that you can set. The only required options are -f, which specifies a file containing a list of fastq files; -t, the transposon tag to search for; and -r, the reference fasta file. To produce the file containing our fastq file, run:

```
$> ls *.fq.gz > fastqs.txt
```

Note that you can specify any number of fastq files here, and these will be run serially. The transposon tag for this sample is TAAGAGACAG. So a complete command to run `bacteria_tradis` on this sample is:

```
$> bacteria_tradis -v --smalt_r 0 -m 0 -f fastqs.txt -t TAAGAGACAG -r
ref.fasta
```

Adding the -v option makes `bacteria_tradis` report its progress while it runs, and can be particularly useful in the case something goes wrong. The --smalt_r 0 and -m 0 options specify that we want to map reads with multiple best mappings to a random position and use these in our downstream analyses; by default these reads are left unmapped (the reasons for this are discussed below under "**Advanced mapping options**"). Mapping and processing this library will take about 30 minutes to an hour on a typical desktop computer.

On completion, `bacteria_tradis` produces a number of files. These include:

(input list name).stats : Mapping statistics file. This is comma delimited, and includes one line for each library mapped along with a header. It can be easily opened in e.g. Excel or R.

(library_name.replicon_name).insert_site_plot.gz : Plot files, one for each replicon and library. These contain insertion counts on each strand for every nucleotide position in the replicon. They can be opened as "user plots" in the Artemis genome browser, and will be used for further analysis.

(library_name).mapped.bam : BAM file containing mapped reads.

If the mapping of `12418_1#10.fq.gz` succeeded, you should now have a file `fastqs.stats` containing one line with mapping statistics for this library. The below screenshot shows this file opened in excel:

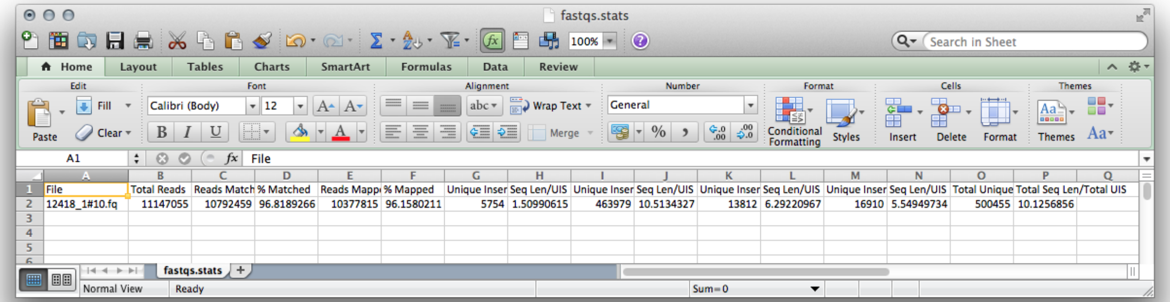

File	Total Reads	Reads Match	% Matched	Reads Mappi	% Mapped	Unique Inser	Seq Len/UIS	Unique Inser	Seq Len/UIS	Unique Inser	Seq Len/UIS	Unique Inser	Seq Len/UIS	Total Unique	Total Seq Len/Total UIS
12418_1#10.fq	11147055	10792459	96.8189266	10377815	96.1580211	5754	1.50990615	463979	10.5134327	13812	6.29220967	16910	5.54949734	500455	10.1256856

Close to 97% of reads had a perfectly matched transposon tag, with 96% of these mapping to the reference replicons, meaning around 93% of sequencing reads in this library are providing useful information. The gzipped plot files can also be opened directly in Artemis (http://www.sanger.ac.uk/science/tools/artemis for download and tutorials). Below is a screenshot showing insertion density over a region of the Salmonella chromosome including two apparently essential genes, *rep*, encoding a DNA helicase, and *rho*, encoding a transcription termination factor:

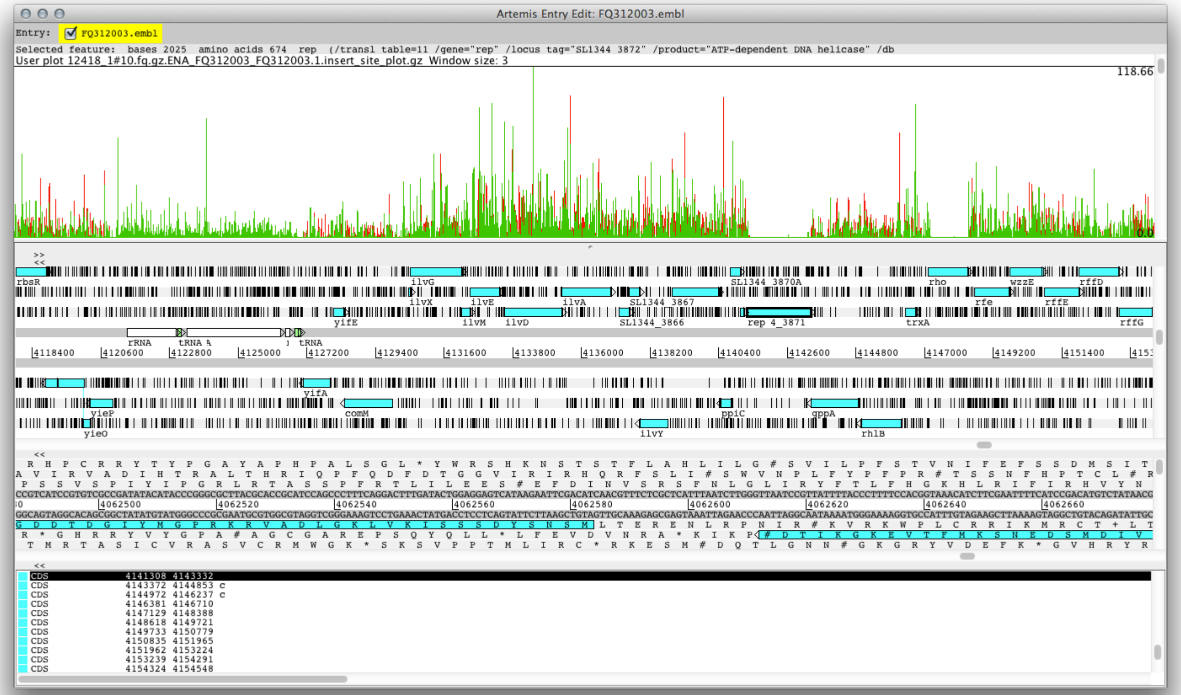

Advanced mapping options

By default, the `bacteria_tradis` pipeline determines appropriate read mapping parameters automatically from the length of the first read in the fastq file. It should be noted that the default parameters have been tested using the optimized TraDIS protocol of Barquist *et al*., 20XX in the hands of an experienced sequencing specialist; these will need to be tuned for other protocols, or for pilot runs, etc. There are various other scenarios in which it would be appropriate to reduce the stringency of these parameters: in the case that read trimming has been applied, if there are quality issues in the library, for certain types of studies (particularly gene essentiality studies as above), or if the quality of the reference genome is low (or of a different strain).

The -mm option specifies the number of mismatches allowed when matching the transposon tag; by default none are allowed. We sometimes observe one or two positions within the transposon tag that seem to have generally low quality. If there is evidence for low-quality

bases in the transposon tag (from FastQC, for instance), setting this to 1 or 2 may result in higher recovery of insertion sites. Higher than 2 is not advisable with the typical transposon tag lengths (10 - 12 bases) produced by TraDIS protocols, but may be appropriate with protocols that produce significantly longer transposon tags.

The -m option sets the minimum mapping quality score to use an alignment in downstream analysis (e.g. plot files); defaults to 30. Multi-mapping reads have a quality score of 0 by definition, so this parameter needs to be set to 0 for these reads to be properly processed. Can be lowered without dramatically affecting results in most cases, particularly if --smalt_y is set reasonably.

The other options specify parameters for the smalt mapper, which are discussed in more detail in the smalt manual (ftp.sanger.ac.uk/pub/resources/software/smalt/smalt-manual-0.7.4.pdf). We will discuss their effects on TraDIS mapping briefly here:

--smalt_k: length of kmers hashed; roughly, the minimum length of an exact match between a read and the genome needed to trigger an alignment attempt. Appropriate values are between ~10 and 20 for bacterial genomes depending on read length. Lower values lead to increased sensitivity at the expense of runtime.

--smalt_s: step size for smalt kmers: distance between the start of hashed kmers. Appropriate values are between 1 and ~15, but should be less than --smalt_k to ensure kmers overlap. Lower values lead to increased sensitivity at the expense of runtime.

--smalt_y: minimum percentage of identical bases between read and reference, defaults to .96 - 96% identity, or 4 mismatches allowed in a 100 base read. May be lowered to improve sensitivity in the case of low quality or short reads.

--smalt_r: specifies what to do with reads that map equally well in multiple locations. By default this is set to -1, meaning that multi-mapping reads are left unmapped. This is appropriate in studies comparing insertion frequency in the same library passaged through multiple conditions, as in this case a change in frequency of one repetitive gene could lead to many genes appearing to be selected artifactually. For studies of gene essentiality in a newly created library, this should be set to 0 (randomly assign a position) to avoid repetitive elements (particularly insertion sequences and the like) artificially appearing to be essential.

Troubleshooting read mapping

Low mapping yields can occur for a number of reasons.

- **Low numbers of tagged reads**: First check in FastQC if there are low quality bases in the transposon tag. If there are, changing -m to 1 or 2 may help. If there is not a quality

issue, and many reads truly lack transposon tags, the sequencing protocol used may not have adequately enriched for transposon-chromosome junctions.

- **Low numbers of mapped reads**: There are several reasons this could occur:
    - **Quality issues or adapter contaminator**: confirm with FastQC, this can often be fixed by trimming and cleaning the reads then relaxing mapping parameters slightly.
    - **Missing replicons**: Does your reference include all replicons for your bacterium, including plasmids? Even small plasmids can eat up a surprising fraction of the read-space if they have high copy numbers; for instance, around 30% of the *Salmonella* reads in our example library originate from plasmids.
    - **Mutant library contamination**: This can happen if multiple transposon libraries are being generated at the same time in the same lab with the same transposon, particularly if batches are being mixed to attain high insertion density. Try mapping against a “meta-reference” containing all the organisms you are working with; if a significant fraction of reads have a best map to a bacterium other than the one you expect, it is likely the sequenced mutant library was contaminated.

Post-processing and analyzing data

For further analysis, we generally process plot files into tab files containing gene-level statistics, which can then be used to perform further analyses, or loading directly into R or Excel. Running:

```
$> tradis_gene_insert_sites -trim3 0.1 FQ312003.embl
12418_1#10.fq.gz.ENA_FQ312003_FQ312003.1.insert_site_plot.gz
```

Will produce a file
`12418_1#10.fq.gz.ENA_FQ312003_FQ312003.1.tradis_gene_insert_sites.csv`
containing locus tags, gene names, read counts and unique insertion sites per gene, among other useful bits of data. The -trim3 option trims reads in the 3′ end of genes, we often use this as many essential genes appear to tolerate insertions towards the end of the coding sequence.

Predicting gene essentiality

To produce an initial prediction of gene essentiality, we can run `tradis_essentiality.R` on the output table:

```
$> tradis_essentiality.R
12418_1#10.fq.gz.ENA_FQ312003_FQ312003.1.tradis_gene_insert_sites.csv
```

This script attempts to fit distributions to the two modes often seen in insertion indexes (that is, insertion sites divided by gene length) in gene essentiality data, see Barquist *et al*., NAR 2013 for more details. This produces tables of putative essential and ambiguous genes, along with

plots that can used to evaluate the predictions. Below is a plot showing the automated fit, along with thresholds for essentiality and ambiguity:

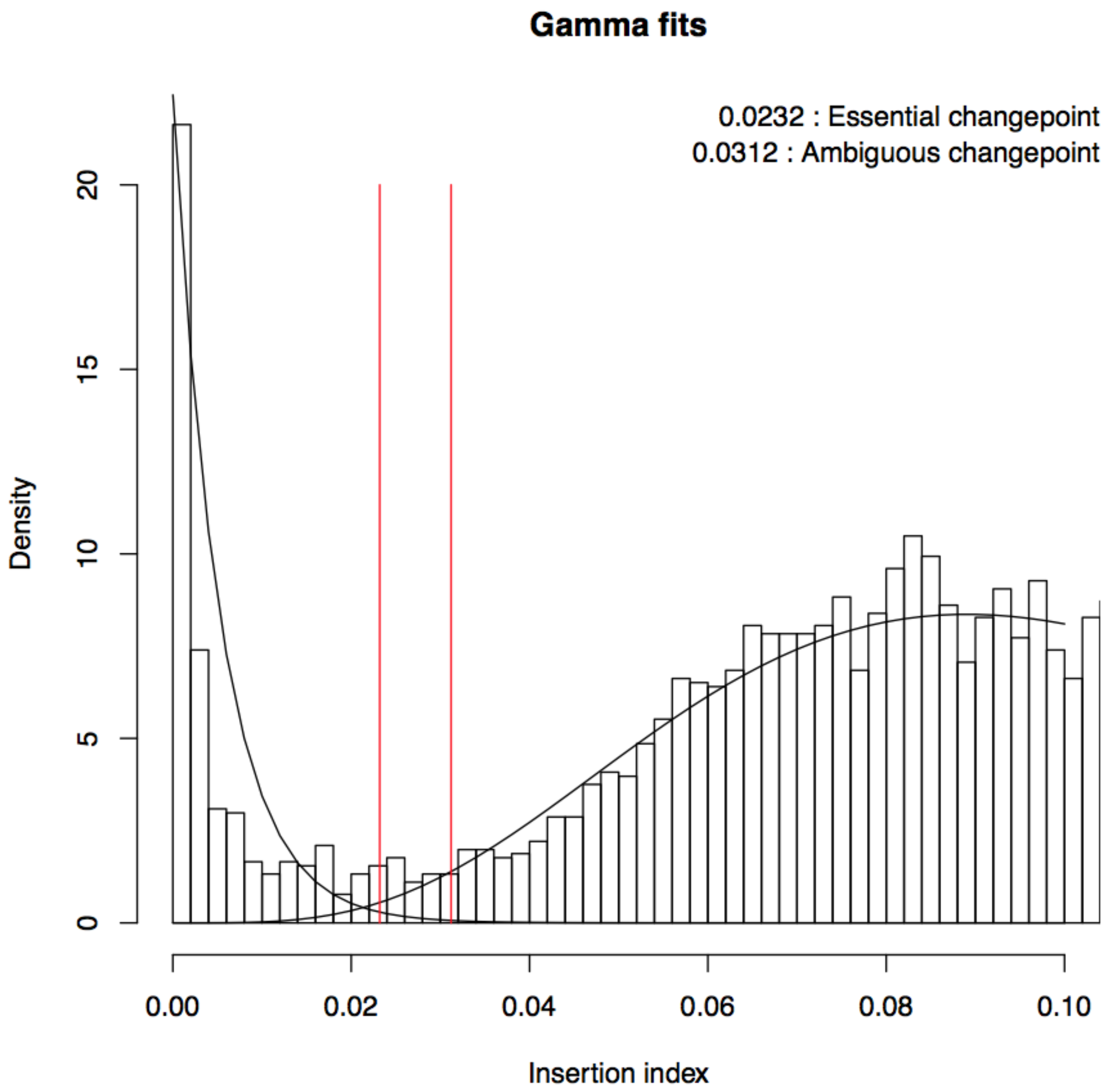

This method predicts 421 genes as essential in our test library, which is comparable to our previous work (Barquist et al., 2013; using hand-fit curves in a closely-related strain) which predicted ~350.

Note that in cases where a library is not sufficiently saturated, the two distributions will collapse to the left, preventing a reasonable prediction of essential genes. An example of this can be seen in the analysis of a *Clostridium difficile* 630 library in Dembek *et al*., mBio 2015. In this case, the best that can be done is to report genes completely lacking insertion sites, with the understanding that some of these will certainly be “false positive”.

Comparing fitness effects

In the case you wish to compare control and test libraries (for instance, a library exposed to an antibiotic as compared to one grown in rich medium), we provide a script `tradis_comparison.R` that uses EdgeR to identify genes with differences in mutant frequency between two conditions, similar to that used in Dembek *et al*., mBio 2015 for the analysis of *C. difficile* sporulation data. Similar to `bacteria_tradis`, it expects files containing lists of tabfiles output by `tradis_gene_insert_sites` for both the control and experimental library. It outputs a number of diagnostic plots, including an MDS plot (similar to a PCA), which can be used to verify that samples cluster on experimental condition rather than some other feature, and volcano plots showing fold-change and -log10(p-values) for every gene. Details on the command-line options available can be found by running it with no arguments.
