# Supplementary material for: The TraDIS toolkit: sequencing and analysis for dense transposon mutant libraries
Source: Bioinformatics. 2016 Jan 21;32(7):1109–11. doi: 10.1093/bioinformatics/btw022 (PMC4896371; doi:10.1093/bioinformatics/btw022)
Supplement: Supplementary Data [file supp_btw022_Tradis_sequencing.pdf]

Contents:

TraDIS adapter and primer design: pages 2 - 4

MiSeq and HiSeq recipe description: pages 5 - 8

Streamlined TraDIS workflow: pages 9 - 11

TraDIS adapter and P7 primer design

Efficient PCR enrichment of transposon/chromosome junctions is critical to the success of TraDIS experiments because only one transposon insertion is expected to occur per genome. In a typical experiment a bacterial genome of 3Mb is fragmented to 300bp during library preparation and on average, only one in every ten thousand fragments will contain a transposon insertion. Effective enrichment of these fragments is, therefore, crucial.

The typical Illumina adapter and primer design strategy generates double stranded fragments containing P5 and P7 sequences (necessary for clustering) by the use of 'Y' shaped adapters (Figure 1A). Using this strategy, the P7 primer hybridises over a much greater region of the adapter than the P5 primer during the first PCR cycle. The first PCR cycle therefore favours the production of fragments with the P7 sequence and not the P5 sequence. Thus, substitution of the typical Illumina P5 primer with a transposon specific primer results in poor enrichment of transposon-containing fragments as products generated from P7 primer binding events dominate. To overcome this we have designed a 'splinkerette' adapter (Rad *et al.,* 2015; Uren *et al*, 2009; Devon *et al*, 1995) and a P7 indexed primer that in principle only hybridises once the transposon-specific primer has generated a transposon-specific complement of the bottom strand (Figure 1B). This results in a strong enrichment for transposon-containing sequences in our TraDIS libraries (see typical qPCR results in Figure 2).

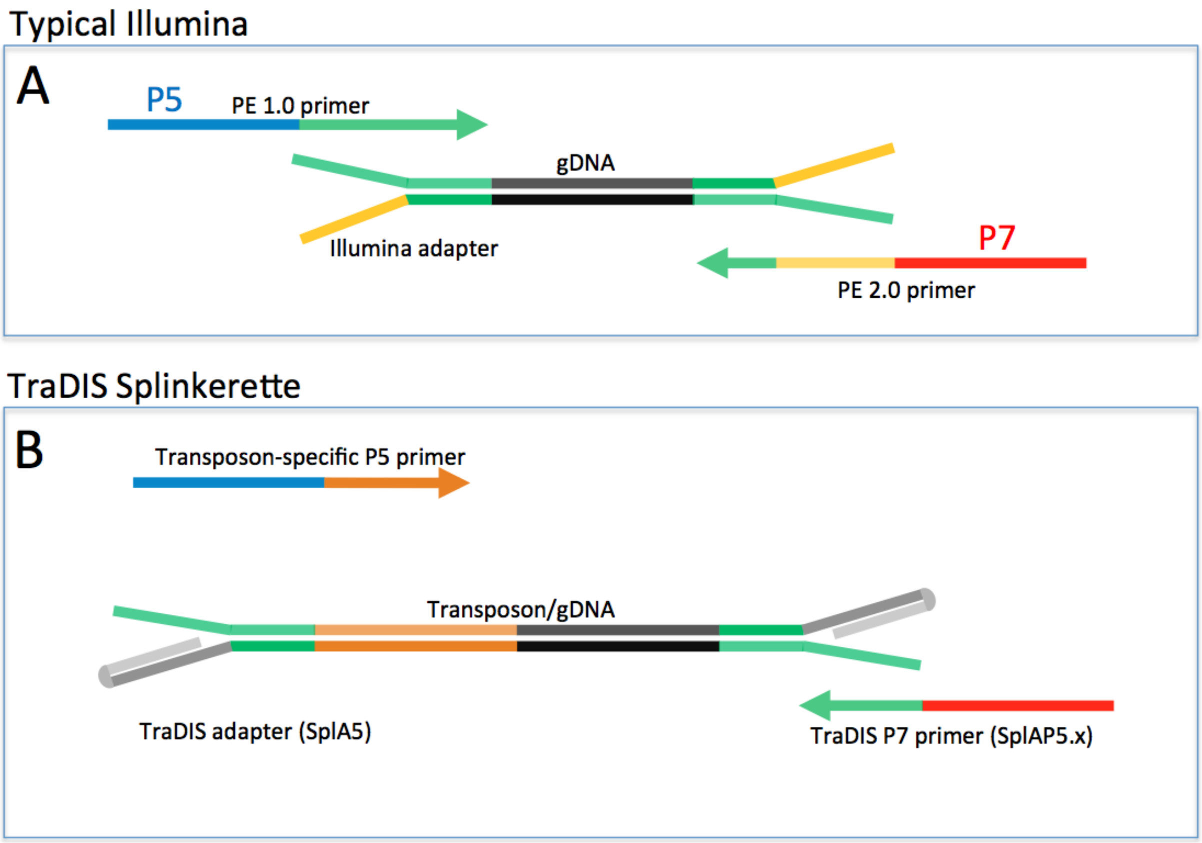

Figure 1. Adapter and primer design strategies for standard Illumina (A) and TraDIS library construction using a splinkerette design strategy (B). Light and dark of the same colour

represent complementary sequence. Transposon and gDNA sequence is represented in orange and black respectively. Adapters are green and grey. The P5 and P7 sequences that are necessary for hybridisation on the flowcell are denoted in blue and red and are incorporated during the PCR enrichment step.

The 'splinkerette' design minimises the production of PCR amplicons that do not contain transposon-gDNA junctions. However, when checking the quality and quantity of these PCR products using a Bioanalyzer (Agilent 2100 High Sensitivity), a characteristic high-molecular weight peak can be observed in the trace (Figure 2). Data from our lab (not shown) suggests that this arises due to interactions between the P7 primer and the adapter-ligated template, and importantly, is unlikely to contain the P5 and transposon sequence necessary for clustering and sequencing. Quantitative evaluation of the main peak using Agilent Bioanalyzer gives comparable data to SYBR green qPCR targeting the P5 and P7 regions. A more specific qPCR using the transposon sequencing primer instead of the P5 primer gives slightly lower data values (Figure 2 and see methods section for detailed protocol). This methodology routinely achieves good clustering densities (800k/mm^2) with typically over 90% of reads containing verified transposon junctions.

A

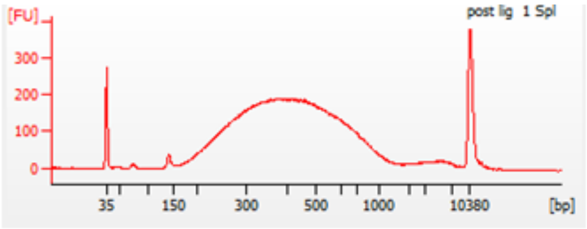

B

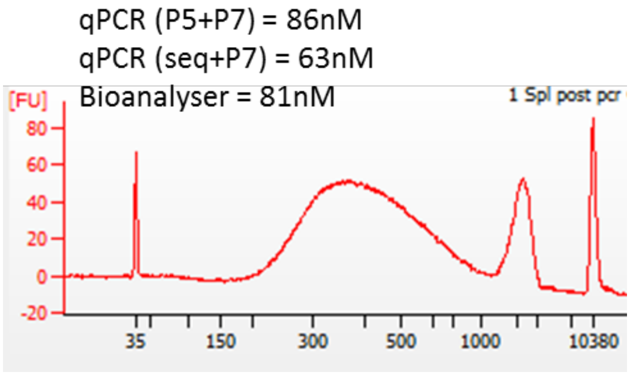

Figure 2. Typical High Sensitivity Agilent Bioanalyzer traces of DNA fragments (1:10 dilution) post-ligation (A) and post-PCR (B) using the TraDIS adapter and primer system. qPCR data was based on SYBR green qPCR using Kapa Illumina standards and primers targeting either the P5 and P7 sequence (P5+P7) or Transposon sequence and the P7 sequence (seq+P7). All qPCR data was normalised to the mean fragment length of 365bp. Bioanalyzer quantification data is of the

main peak only. The high molecular weight peak (>1000bp) probably arises due to interactions between the P7 primer and the template (data not shown)

1. Transposon primer design for PCR and sequencing

In order to increase the specificity, robustness and reproducibility of TraDIS experiments, the transposon enrichment primer is designed to be located upstream (5') of the sequencing primer binding site (Figure 3). The 3' end of the transposon-specific sequencing primer is designed to hybridise exactly 10bp upstream of the transposon/chromosome junction (Figure 3). During sequencing this gives 10 bp of transposon sequence that can be used as verification that a read is directly adjacent to the expected transposon inverted repeat.

Primer melting temperature (Tm) is an important parameter to consider, particularly for the sequencing primers, which need to be similar to the Tm of Illumina's read 1 sequencing primer (77°C when using Sigma OligoEvaluator on the default settings: http://www.oligoevaluator.com/Login.jsp). If the sequencing primer Tm is significantly lower (greater than 8°C below Illumina read 1 Tm), then cluster intensity will drop, resulting in poor quality base calling. Similarly, the transposon PCR enrichment primers are designed to have a similar Tm to that of the corresponding Illumina primers (PE1.0 and PE2.0). This enables the same PCR cycling protocol to be used for all transposons (see Methods section). In addition, it is important to achieve a Tm of greater than 64°C (using Sigma OligoEvaluator on default settings) for the portion of the enrichment primer that hybridises to the transposon during the first PCR cycle. Adapter and primer sequences can be found in the "recipes" directory of the Bio-Tradis git archive.

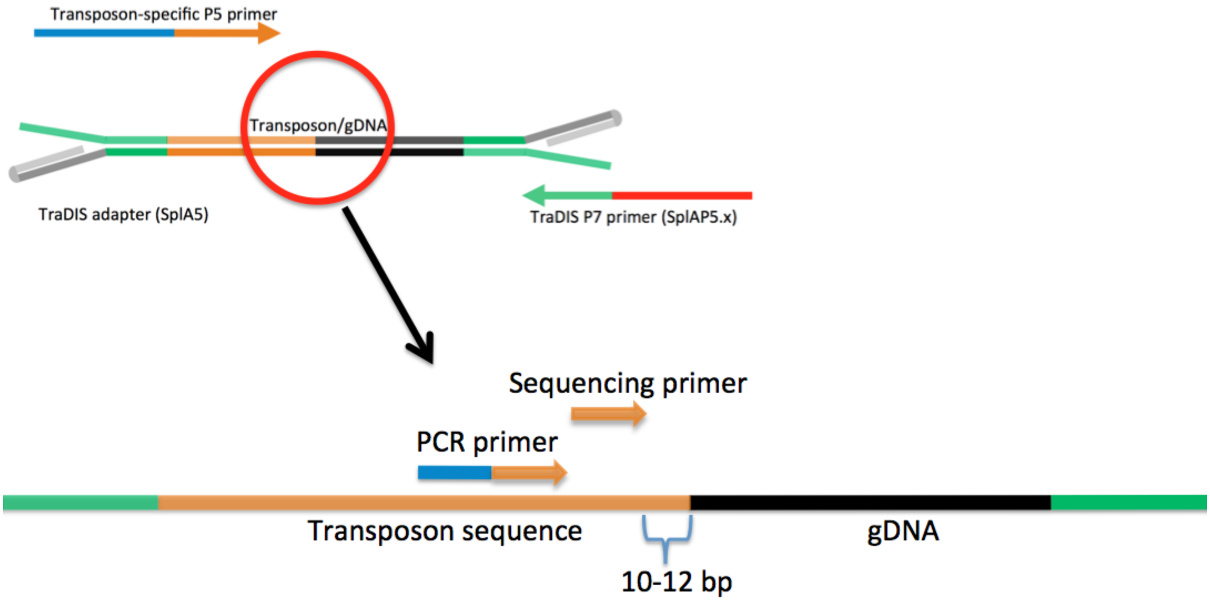

Figure 3. Primer design strategy for TraDIS sequencing. Colours and descriptions are as in Figure 1. The transposon-specific enrichment PCR primer hybridises to the transposon sequence upstream (5') of the sequencing primer binding site, although some overlap is tolerated. The transposon-specific sequencing primer hybridises 10bp 5' of the junction with gDNA.

MiSeq and HiSeq 2500 recipes

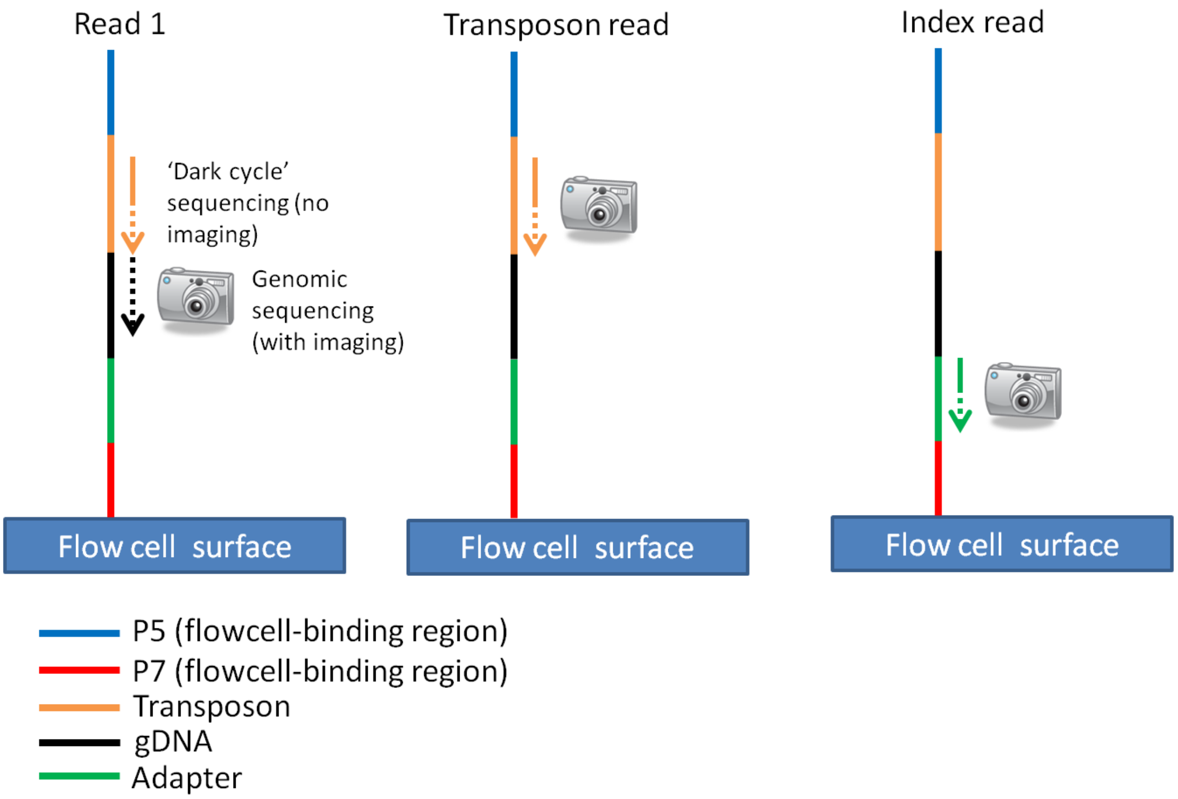

Figure 4. HiSeq and MiSeq TraDIS recipes allow for 'dark' sequencing across the difficult monotemplate sequence of the transposon. The transposon-specific sequencing primer hybridises to the known transposon sequence 10 bp upstream of the junction with gDNA. Sequencing takes place with no imaging for 10 or 12 cycles (10 or 12 for MiSeq, 12 for Hiseq2500) and continues with imaging for 42 cycles. The transposon sequence is generated as a separate 10bp (MiSeq) or 12bp (HiSeq2500) read following read 1 and before the index read.

Although TraDIS libraries could potentially be run on the MiSeq and HiSeq2500 machines using standard Illumina recipes, the low complexity sequence in the first part of the insert can cause problems with focusing and template generation unless there is a high amount of a more complex 'standard' library such as PhiX pooled with it. We therefore use a dedicated custom TraDIS recipe incorporating 'dark' cycles (figure 4). The MiSeq protocols includes 10 or 12 cycles of dark sequences, while the HiSeq protocol includes 12 cycles of dark sequencing. Either of the 12 dark cycle recipes would be appropriate for use with transposons with biased integration sites, such as Mariner/Himar1 transposons, with a 10-base tag. These are run on the MiSeq with 5% PhiX and HiSeq 2500 (rapid mode) with 10% PhiX. Both these recipes are downloadable and need to be transferred across to the respective machines. Note that

neither recipe is formally supported by Illumina. These recipes are freely available for download from https://github.com/sanger-pathogens/Bio-Tradis in the "recipes" directory.

- Transfer the MiSeq 'Transposon10' or 'Transposon12' recipe folder to the V2 subfolder (Computer – C – Illumina – MiSeq Control software – recipe – V2)
- Transfer the HiSeq2500 'Tradis_SR_RR_12dark_42r1_rehyb12_index8.xml' recipe file to Recipe folder (C – Illumina – HiSeq Control Software – Recipe)

Running the HiSeq2500

For a HiSeq2500 run choose the 'Tradis_SR_RR_12dark_42r1_rehyb12_index8' recipe during run configuration – tick 'Existing Recipe' and then navigate to the location where the recipe was saved (see Figure 5 below).

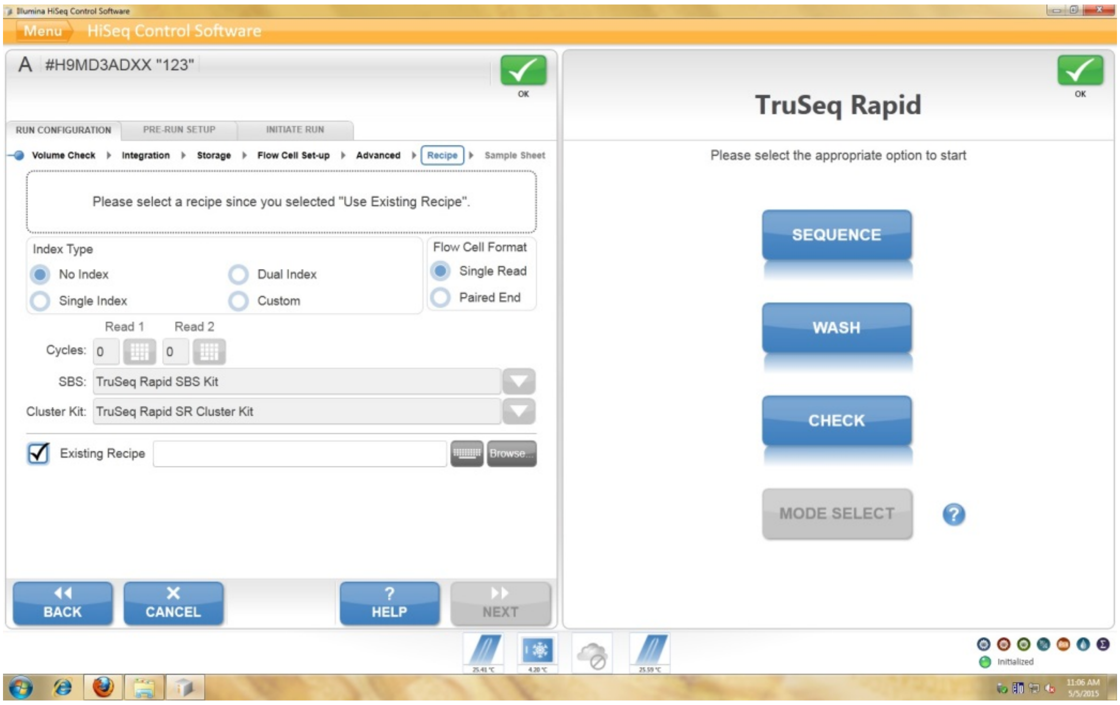

Figure 5. Screenshot showing the step during HiSeq2500 setup when the custom recipe can be selected.

Spike in 37.5µl (100µM) of each TraDIS custom primer into 2ml of HT1 and then add this to the HP10 tube (already containing Illumina Read1 primer). Add 10µl (100µM) iPCRtagseq index read sequencing primer into the HP12 tube.

Note that for the HiSeq2500 recipe the number of cycles is set by the recipe at 42 cycles of DNA and this starts at base 13 (i.e. after the 12 dark cycles). This should be sufficient for mapping to bacterial genomes and allows a 50 cycle kit to be used. For a TraDIS run of 42 cycles the actual number of sequencing cycles would be 74 cycles including 8 index cycles – a 50 cycle kit contains enough reagents for up to 75 cycles of sequencing.

Running the MiSeq

The MiSeq uses information contained within the sample sheet .csv file to set up, perform and analyse a sequencing run. To run the custom TraDIS recipe on the MiSeq a 'standard' sample sheet is modified so that the correct chemistry is used ('Transposon10' or 'Transposon12'), the expected transposon tag sequence is added as the first index sequence, and the true index sequence as index2 (see example below). Note that the number of transposon tag sequencing cycles is dictated by the expected transposon sequence entered into the sample sheet. The number of gDNA sequencing cycles can be changed but for a 50 cycle MiSeq kit the total number of cycles must not exceed 75 cycles – effectively, for a 10 base transposon tag and an 8 base index tag, this limits the number of gDNA cycles to a maximum of 47 cycles.

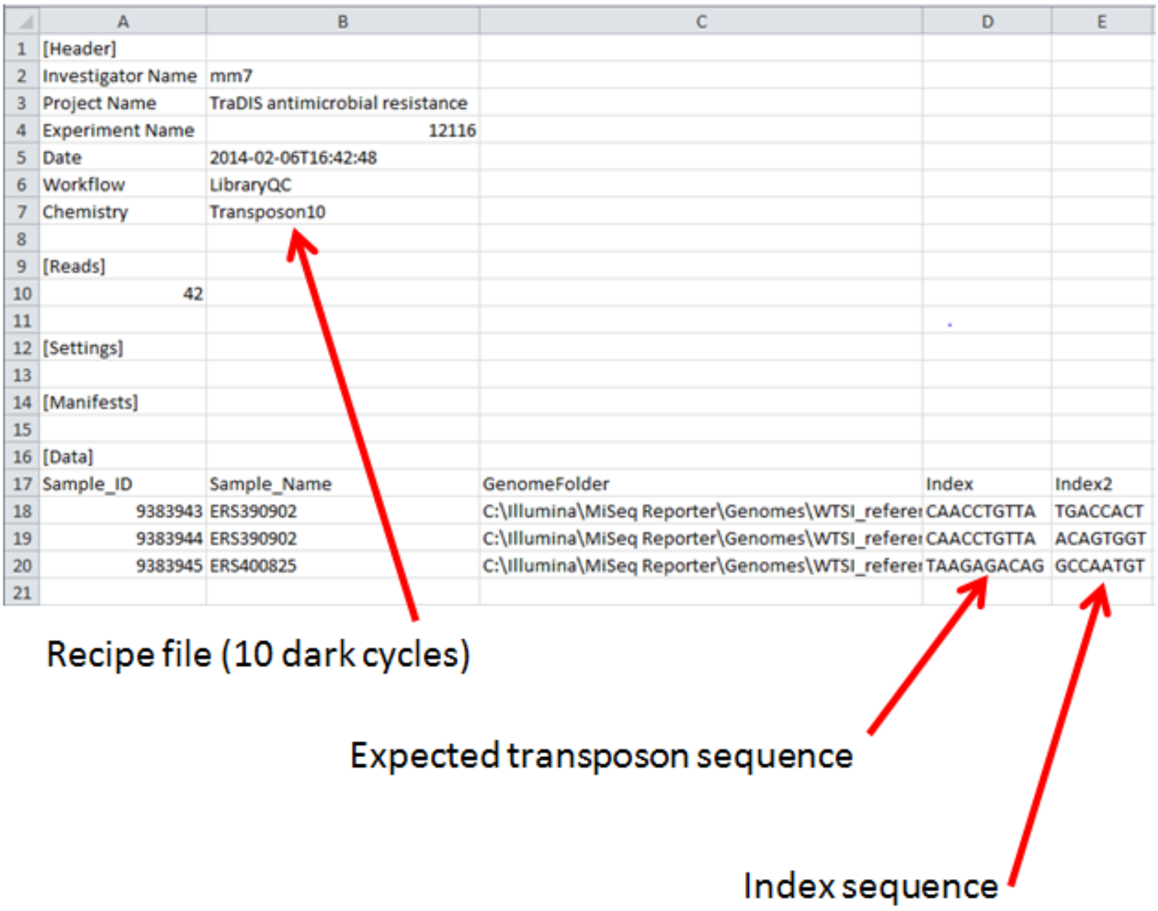

	A	B	C	D	E
1	[Header]				
2	Investigator Name	mm7			
3	Project Name	TraDIS antimicrobial resistance			
4	Experiment Name	12116			
5	Date	2014-02-06T16:42:48			
6	Workflow	LibraryQC			
7	Chemistry	Transposon10			
8					
9	[Reads]				
10	42				
11					
12	[Settings]				
13					
14	[Manifests]				
15					
16	[Data]				
17	Sample_ID	Sample_Name	GenomeFolder	Index	Index2
18	9383943	ERS390902	C:\Illumina\MiSeq Reporter\Genomes\WTSI_referer	CAACCTGTTA	TGACCACT
19	9383944	ERS390902	C:\Illumina\MiSeq Reporter\Genomes\WTSI_referer	CAACCTGTTA	ACAGTGGT
20	9383945	ERS400825	C:\Illumina\MiSeq Reporter\Genomes\WTSI_referer	TAAGAGACAG	GCCAATGT
21					

Figure 6. Example sample sheet for a MiSeq TraDIS run. The essential changes compared to a standard run are indicated by the red arrows.

The TraDIS library is denatured and loaded according to standard Illumina protocols. 4µl of 100µM transposon-specific sequencing primer plus 4µl of 100µM Illumina read1 sequencing primer are added to 600µl buffer HT1 and this goes into port 18 of the MiSeq reagent cartridge. 4µl of index read primer (iPCRtagseq) at 100µM is added to 600µl of the HT1 buffer and this goes into port 19.

Streamlined TraDIS workflow

To allow the TraDIS method as described in Langridge *et al* (2009) to be automated, and to increase yields generated from the input DNA, we replaced Qiagen column clean-up steps with Ampure bead purification and dispensed with the post-ligation agarose gel size-selection step (figure 1). A more limited size selection is imposed by the use of Ampure SPRI beads at 0.8:1 ratio which removes library fragments below 200bp in size. Bias caused by uneven PCR amplification of different sized fragments should not occur because any particular transposon/chromosome junction will be represented by sequence data generated from a range of fragment sizes (since the initial fragmentation by sonication is random). Moreover use of Kapa Hifi during PCR ensures even amplification of fragments with a range of GC content (Quail *et al*, 2012. Nature Methods).

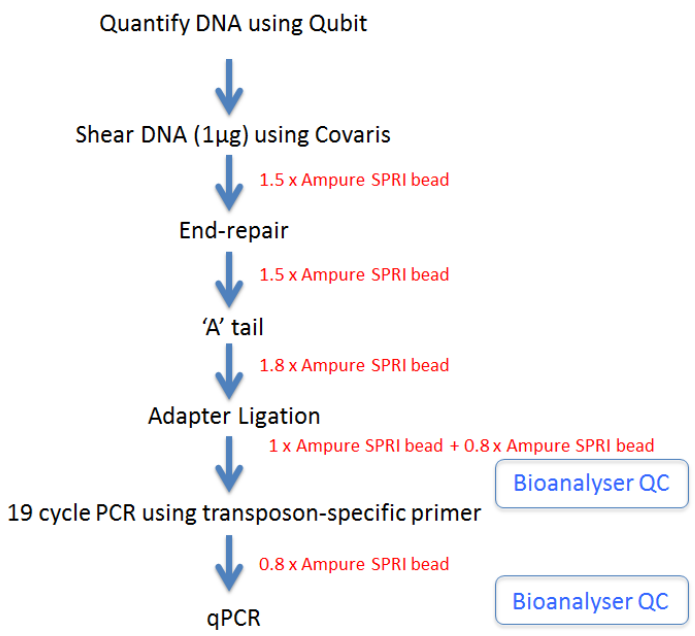

Figure 7. Automation-friendly workflow for TraDIS library construction.

Manual TraDIS library protocol

The TraDIS library construction protocol is based on our general library prep methodology (see 'Improved Protocols for Illumina Sequencing', Bronner *et al*, 2013), except for the use of a TraDIS-specific adapter (SplA5) and primers (Transposon-specific primer and indexed adapter-specific SplAP5.x primer (Integrated DNA Technologies).

For both transposon ends to be sequenced, 2µg of high quality genomic DNA, quantified using a Qubit fluorometer (Lifetechnologies), was sheared to ~300 bp fragments using

Covaris' Adaptive Focused Acoustics (AFA) technology using 6 mm × 16 mm AFA fiber minitubes (Covaris, cat. no. 520045) with 8mm Crimp-Caps (Cat. No. 520028). The settings routinely used on the Covaris S2 or E210 instruments were as follows: Duty cycle, 20%; Intensity, 5; Cycles, 200; Time, 60s.

Following Covaris shearing a 1.5 x Ampure SPRI (Beckman Coulter Genomics) clean-up was performed following the manufacturers protocol and this was eluted with 50 µl Elution buffer (EB).

The fragments were then end repaired using the NEBNext End Repair module (NEB, cat. no. E6050) following the manufacturer's instructions and subsequently purified with a 1.5x Ampure SPRI clean-up with elution in 32µl EB buffer.

'A' tailing was carried out using the NEBNext dA-Tailing Module (NEB, cat. no. E6053) following the manufacturer's instructions and this was followed by a 1.8x Ampure SPRI clean-up with a final elution in 20µl EB buffer.

All 20µl of 'A' tailed DNA was used in the adapter ligation reaction. For this the NEBNext Quick Ligation Module (NEB, cat. no. E6056) was used following the manufacturers protocol except for the use of 1µl of 100µM SplA5 adapter per sample reaction. Following adapter ligation a 1x Ampure SPRI clean-up was performed with elution in 50µl EB followed by a 0.8x Ampure SPRI clean-up with elution in 21µl. A 1µl aliquot of this was then diluted 1 in 10 in water and resolved on an Agilent Bioanalyzer using a High Sensitivity DNA kit (Agilent Technologies). A typical profile consisted of a broad peak in the 300 – 500bp range with small amounts of adapter (75bp) and adapter dimer (140bp) usually being present.

PCR enrichment of fragments containing the transposon sequence was carried out using the transposon-specific primer (see supplementary material) and an indexed adapter-specific primer (SplAP5.x). PCR was carried out using Kapa HiFi HotStart ReadyMix (Kapa Biosystems, cat. no. KK2601) with a typical reaction mixture as follows:

Adapter-ligated DNA	10 µl (should be at least 100ng)
Kapa HiFi 2x ReadyMix	25 µl
5' or 3' transposon specific primer (100µM)	0.5 µl
SplAP5.x index primer (10µM)	5 µl
Water	9.5 µl

The PCR cycling conditions used were as follows:
95˚C for 2 minutes, then 19 cycles of 98˚C for 20 seconds, 65˚C for 30 seconds, 72˚C for 30 seconds, and a final extension step of 72 ˚C for 2 minutes.
The PCR products were then purified using a 0.8x Ampure SPRI clean-up with elution in 30µl EB buffer. Note that the post-ligation eluted volume of 21µl is sufficient for PCR enrichment of both transposon ends. If only one transposon end was required 1µg of gDNA was used for library preparation and 20µl of the post-ligation DNA was used as template for the PCR.
A 1µl aliquot of the PCR products was then diluted 1 in 10 in water and resolved on an Agilent Bioanalyzer using a High Sensitivity DNA kit (Agilent Technologies). A typical profile usually consisted of a broad peak in the 350 – 500bp range with no primer or

primer dimer evident. Occasionally a pronounced peak around 2kb in size was observed but this was ignored (see Supplementary material, Figure 2).

Although the Bioanalyzer is good for a qualitative assessment of these libraries we have found it to be unreliable for quantitative assessment of TraDIS libraries. For this reason SYBR green qPCR was used (Kapa Biosystems, cat. no. KK4824), albeit with the use of alternative primers (see below). Routinely two qPCRs were performed per sample with one utilising primers binding the P5 and P7 sequences necessary for cluster formation and the other binding the transposon sequence (the sequencing primer) and P7 sequence. This allows a distinction to be made between fragments that will cluster and those that will give transposon containing reads. Transposon-containing fragments were typically quantified as 40-90% of the total fragments that could potentially cluster. Note that because the sequencing primer binds a transposon region downstream of where the transposon-specific PCR binds, sequenced reads tended to be almost entirely true transposon-genomic DNA junctions (>90%). The 20µl qPCR reactions were set up as follows:

<u>P5+P7 qPCR</u>

DNA (diluted 1:1000 or 1:5000))	4µl
Kapa SYBR FAST 2x mix	10µl
10µM qPCR2.1 primer	0.4µl
10µM qPCR2.2 primer	0.4µl
Water	5.2µl

<u>Transposon+P7 qPCR</u>

DNA (diluted 1:1000 or 1:5000))	4µl
Kapa SYBR FAST 2x mix	10µl
10µM Transposon sequencing primer	0.4µl
10µM qPCR2.2 primer	0.4µl
Water	5.2µl

Note that for the transposon-specific qPCR the transposon-specific sequencing primer was used.

Two master mixes were always made up. For the P5- and P7-targeting reactions the quantity of master mix was increased so as to be sufficient for the six Kapa standards. The qPCR data generated from the transposon sequencing primer qPCR reactions were used to determine MiSeq or Hiseq2500 loading concentration with loading at 8pM generally being optimal.
